# Aging-dependent YAP1 reduction contributes to AD pathology by upregulating the Nr4a1-AKT/GSK-3β axis

**DOI:** 10.1186/s40035-025-00487-4

**Published:** 2025-06-04

**Authors:** Ling Lei, Yilei Cheng, Anqi Yin, Jian-Min Han, Gang Wu, Fumin Yang, Qi Wang, Jian-Zhi Wang, Rong Liu, Hong-Lian Li, Xiaochuan Wang

**Affiliations:** 1 Hubei Key Laboratory of Cognitive and Affective Disorders, School of Medicine, Jianghan University, Wuhan, 430056 China; 2 School of Basic Medicine, Key Laboratory of Education Ministry/Hubei Province of China for Neurological Disorders, Tongji Medical College, Huazhong University of Science and Technology, Wuhan, 430030 China; 3 Wuhan Sixth Hospital Affiliated to Jianghan University, Wuhan, 430015 China; 4 Co-Innovation Center of Neuroregeneration, Nantong University, Nantong, 226001 China

**Keywords:** YAP1, Nr4a1, Alzheimer’s disease, Senescence

## Abstract

**Background:**

Aging is the greatest risk factor for late-onset Alzheimer’s disease (LOAD), which accounts for > 95% of all Alzheimer’s disease (AD) cases. Yes-associated protein 1 (YAP1), an aging-dependent protein, is a key element in the classical Hippo-YAP1 pathway mediated by a kinase cascade. Research showed that YAP1 was markedly reduced in the brains of individuals with AD. However, the mechanisms underlying the susceptibility of the Hippo-YAP1 signaling pathway in the context of LOAD remain unclear.

**Methods:**

AAV9-YAP1-RNAi was injected into the hippocampi of C57BL/6J mice to establish a YAP1 knockdown model. Overexpression of full-length YAP1 was achieved by injecting AAV9-YAP1 into the hippocampi of SAMP8 mice. To establish the model of knockdown of nuclear receptor subfamily 4 group A member 1 (Nr4a1), AAV9-Nr4a1-RNAi was injected into the hippocampi of SAMP8 mice. In the C57BL/6J mice with YAP1 knockdown, Nr4a1 expression was either knocked down or inhibited with DIM-C to examine the impact of Nr4a1 on tau phosphorylation and cognitive deficits. Primary hippocampal neurons from Sprague–Dawley (SD) rats were infected with lentivirus (LV)-YAP1 to create a YAP1 overexpression model, and Aβ treatment was used to induce neuronal senescence. Protein levels were assessed using immunofluorescence, Western blotting, and ELISA. Animal behavior was evaluated using the Morris water maze test, novel object recognition test, and open field test.

**Results:**

YAP1 was reduced in the hippocampus of both aged C57BL/6J mice and SAMP8 AD model mice through Hippo pathway activation, as well as in Aβ-induced senescent neurons. Overexpression of YAP1 in primary neurons significantly mitigated the Aβ-induced neuronal senescence by downregulating several senescence-related genes, including p16 and p53. The levels of phosphorylated AKT/GSK-3β in neurons were increased with overexpression of YAP1 both in vivo and in vitro. Knockdown of YAP1 induced AD-like symptoms and exacerbated cognitive decline in 2-month-old C57BL/6J mice. Injection of AAV9-YAP1 in the brains of SAMP8 mice partially alleviated neuronal senescence and enhanced cognitive function. Notably, genetic knockdown and chemical inhibition of Nr4a1 significantly ameliorated cognitive deficits as well as AD-like pathology in these subjects.

**Conclusions:**

These findings reveal an etiopathogenic relationship between aging and AD, which is associated with the YAP1-Nr4a1-AKT/GSK-3β signaling pathway. Our findings provide insight into the therapeutic strategies aimed at delaying brain aging and combating neurodegenerative diseases such as AD.

**Supplementary Information:**

The online version contains supplementary material available at 10.1186/s40035-025-00487-4.

## Background

Alzheimer’s disease (AD) is a neurodegenerative disorder closely associated with aging and a major cause of dementia in the elderly [1], affecting over 30 million individuals worldwide. Currently, there is no effective treatment for AD, largely due to incomplete understanding of its etiology and pathogenesis [2, 3]. It is estimated that the incidence of AD doubles every 5 years after age 65, and 50% of the population aged 85 or older suffer from AD. Therefore, aging is considered the greatest risk factor for AD, although the mechanisms are not well understood. Evidence from human and animal studies indicates that cellular senescence plays a key role in the development of many aging-related diseases [4–8], including AD [9–12]. Senile plaques composed of extracellular β-amyloid (Aβ) deposits, and neurofibrillary tangles (NFTs) formed by intracellular accumulation/deposition of hyperphosphorylated tau proteins, are two neuropathological features of AD [13, 14]. Although it is still on debate whether and how Aβ and hyperphosphorylated tau lead to neurodegeneration, accumulating evidence indicates that both Aβ and tau pathologies are potent inducers of cellular senescence. Senescent neurons have been detected in the brains of AD patients [3, 9, 11, 12, 15, 16] and AD model mice that overexpress Aβ or tau protein [10, 11, 17, 18]. Removal of senescent cells using pharmacological and genetical approaches reduces brain Aβ load and tauopathy and improves memory in AD model mice [10, 11, 17, 18]. Senescent cells typically exhibit distinct morphological changes and elevated levels of markers such as SA-β-Gal, p21, and p16 [19, 20]. It has been reported that the level of p16 in pyramidal neurons from the prefrontal cortex of elderly humans is significantly higher compared to that in younger individuals. [21]. Single-cell RNA sequencing of postmortem brain tissues from 76 individuals with varying stages of AD pathology detected approximately 2% senescent cells from ~ 140,000 single nuclei, with more than 97% of the senescent cells being neurons, and these findings were strongly correlated with the presence of tau protein NFTs [22]. These data strongly support the hypothesis that cellular senescence mediates the neuropathophysiology induced by Aβ and tau in AD.

The Hippo signaling pathway is a key regulator of stem cell self-renewal, tissue regeneration, and organ size, and is a conserved, classical pathway mediated by a kinase cascade [23–25]. When activated, the Hippo signaling pathway triggers the phosphorylation of Yes-associated protein 1 (YAP1) by the upstream kinases Lats1 and Lats2 at serine site 127 (S127), leading to the binding of the phosphorylated YAP1 to 14–3-3 proteins. This interaction sequesters YAP1 in the cytoplasm, promoting its degradation and inactivation. Conversely, when the Hippo pathway is inactivated, the phosphorylation cascade does not occur, so the unphosphorylated YAP1 can translocate into the nucleus, where it binds to the transcriptional coactivator TEAD [26]. As a key effector of the Hippo signaling pathway, YAP1 plays a critical role in regulating cell proliferation, differentiation, and tissue regeneration [27–29]. Recent studies have highlighted the involvement of YAP1 signaling in aging and cellular senescence [30–33]. YAP1 deficiency has been shown to promote healthy aging in *Caenorhabditis elegans* [31], while YAP1 inhibits cellular senescence in human fibroblasts [33, 34], mesenchymal stem cells [30], periodontal ligament stem cells [32], glioma cells [35], and hepatic stellate cells [36]. A comprehensive analysis of brain expression profiles indicates that YAP1 acts as an important upstream regulator in AD [37]. However, the molecular mechanisms by which YAP1 regulates AD pathology remain unclear. Some studies suggest that YAP1 may negatively regulate the expression of nuclear receptor subfamily 4 group A member 1 (Nr4a1) [38, 39]. Nr4a1 (also called Nur77, TR3, or NGFIB) is an orphan nuclear receptor that can be rapidly and transiently induced by various stimuli [40, 41]. Earlier studies have reported increased Nr4a1 expression in postmortem brain tissues from AD patients [42, 43]. Nevertheless, it remains unclear whether YAP1 contributes to AD-like lesions by modulating Nr4a1 expression in the aging brain and in AD.

This study aims to comprehensively investigate the precise mechanisms by which YAP1 downregulation contributes to pathological alterations in AD brains. Furthermore, it seeks to determine whether YAP1 upregulation can mitigate or ameliorate age-related neurodegenerative conditions and AD-like pathological characteristics. The research results will probably provide valuable scientific insights into developing therapeutic strategies and identifying potential targets for age-related neurodegenerative disorders.

## Materials and methods

### Animals

Male SAMP8 mice (P8), SAMR1 mice (R1), and C57BL/6J mice were obtained from the Institute of Genetics and Developmental Biology at the Chinese Academy of Sciences (Beijing, China). Animals were kept on a 12 h light/dark cycle with free access to food and water.

### Stereotaxic injection

Mice were anesthetized with isoflurane vaporizer (2%–4%) and positioned in a stereotactic frame (RWD, Shenzhen, China). The skull was exposed and aligned with bregma as the reference point. Using a Hamilton syringe, adeno-associated virus (AAV) was injected bilaterally in the hippocampal region at the following coordinates relative to bregma: ± 2.1 mm (M/L), + 1.9 mm (A/P), and − 2.1 mm (D/V). A total of 10^13^ viral genomes (vg) were injected on each side at a flow rate of 0.5 µL/min. After completing the injections, syringes were left in place for 8 min to allow for complete diffusion of the viral solution before suturing the incision site. The mice were monitored until recovery from anesthesia was confirmed. Following a period of 30 days post-injection, behavioral testing was conducted prior to euthanizing the mice for subsequent biochemical analyses.

### Primary neuron culture, lentiviral infection and neuronal senescence induction

Primary rat hippocampal neurons were isolated from rat embryos at embryonic day 17 or 18 and subsequently cultured in neurobasal (Gibco, Invitrogen; Bleiswijk, Netherlands, #21103049) supplemented with 1 × B27 (Gibco, #17504044), 1 × GlutaMAX, and 100 U/mL penicillin–streptomycin (Gibco, #15140122) in a mixed culture solution. Cells were maintained in a humidified incubator at 37 °C with an atmosphere of 5% CO_2_. Pregnant Sprague–Dawley rats were from the Laboratory Animal Center of Tongji Medical College, Huazhong University of Science and Technology, where they were raised under standardized laboratory conditions.

The cultured neurons were infected with lentiviral vectors at five days in vitro (DIV5). Neuron senescence model was established by Aβ, based on a previously described method with minor modifications [19]. In brief, neurons at DIV7 to DIV10 were cultured in dishes coated with poly-*D*-lysine. The culture medium was maintained as previously described but supplemented with 5 μmol/L Aβ, after which the cells underwent an additional incubation period of 24 h [44]. During this incubation, cells were closely monitored for morphological changes indicative of senescence, and the experiments were performed in multiple wells for each group to ensure experimental reliability.

### Viruses and reagents

The initial construction, processing, and packaging of AAV9-YAP1 (hSyn promoter-EGFP-MCS-SV40 PolyA), AAV9-YAP1-RNAi (hSyn promoter-EGFP MIR155(MCS)-WPRE-SV40-PolyA), AAV9-Nr4a1-RNAi (U6-MCS-CAG-EGFP), and LV-YAP1 (Ubi-MCS-3FLAG-CBh-gcGFP-IRES-puromycin) were performed by Shanghai Gene Chemistry Co., Ltd. Aβ1-42 peptide was synthesized by Shanghai Chinapeptides Biotechnology Co., Ltd. with a purity exceeding 95%, and the lyophilized powder was stored at − 80 °C. Before use, the powdered compound was dissolved in dimethyl sulfoxide (DMSO) to ensure complete solubilization. Then the solution was carefully diluted in an appropriate culture medium to achieve working concentrations. The diluted solution was then incubated overnight at a controlled temperature below 4 °C to facilitate aggregation of Aβ into oligomers. DIM-C-pPhOH (HY-112055, MedChemExpress) was dissolved in DMSO to yield an effective stock solution at a concentration of 12.5 mg/mL. The DIM-C-pPhOH solution in DMSO was injected into the abdominal cavity of mice at a dosage of 50 mg/kg [45], while another portion was diluted in corn oil at a ratio of 1:10 to obtain a final concentration of 1.25 mg/mL for oral administration to mice. C57BL/6J mice aged three months received either vehicle (2% DMSO in corn oil; 10 µL/g body weight) or DIM-C-pPhOH (50 mg/kg in 2% DMSO/corn oil; 10 µL/g body weight) once daily over one week.

### Western blotting

The primary antibodies used for Western blotting [46] were rabbit anti-YAP1 (A1002 and A21216, ABclonal, Wuhan, China, 1: 1000), rabbit anti-p-YAP1 (13008S, CST, Danvers, MA, 1:1000), rabbit anti-Nr4a1 (ab153914, Abcam, Cambridge, UK, 1:1000), rabbit anti-LATS1 (A17992, ABclonal, 1:1000), rabbit anti-p-LATS1 (ser909) (#9157, CST, 1:1000), rabbit anti-MST1 (#A8043, ABclonal, 1:1000), rabbit anti-p-MST1/2 (Thr183/Thr180) (AP1094, ABclonal, 1:1000), mouse anti-p-AKT (Thr308) (sc-271966, Santa Cruz, Dallas, TX, 1:1000), mouse anti-AKT (40D4, CST, 1:1000), rabbit anti-p53 (60,283–2-Ig, Proteintech, Suite, Rosemont, IL, 1:1000), rabbit anti-GSK3β (A11731, ABclonal, 1: 1000), rabbit anti-GSK3β (Ser9) (AP0039, ABclonal, 1:1000), mouse anti-AT8 (Ser202.Thr205) (MN1020, Invitrogen, 1:1000), mouse anti-PSD95 (ZMS1068, Sigma, St. Louis, MO, 1:1000), rabbit anti-Synaptophysin (17785–1-AP, Proteintech, 1:1000), mouse anti-p16 (sc-1661, Santa Cruz, 1:1000). Rabbit anti-β-actin (AC026, ABclonal, 1:10,000), mouse anti-tau5 (ab80579, Abcam, 1: 1000) and rabbit anti-tau5 (R25863, Zen-Bioscience, Chengdu, China, 1: 1000) were used as a loading control. Immunoreactive bands were visualized using the Odyssey Infrared Imaging System. Band intensity quantitation was performed using the ImageJ (Fiji) software.

### Immunofluorescence assay

Immunostaining of tissue sections was performed as previously described [47]. The primary antibodies included rabbit anti-YAP1 (A1002 and A21216, ABclonal, 1:200), mouse anti-AT8 (MN1020, Invitrogen, 1:200), rabbit anti-Aβ (#8243, CST, 1:200), mouse anti-IBA1 (sc-32725, Santa Cruz, 1:200), rabbit anti-IBA1 (#17,198, CST, 1:200), mouse anti-GFAP (#3670, CST, 1:200), rabbit anti-NeuN (#24307, CST, 1:200), mouse anti-NeuN (ab177487, Abcam, 1:200), mouse anti-p16 (sc-1661, Santa Cruz, 1: 200), and rabbit anti-p16 (#18769, CST, 1: 200). Following three additional PBS washing steps to remove unbound portions and minimize background staining, mounting was completed. Image acquisition was performed using a confocal microscopy (LSM710, Zeiss, Oberkochen, Germany). The resulting images were processed and analyzed with the ImageJ software to quantify fluorescence intensity.

### SA-β-gal staining

Cellular senescence was assessed using the SA-β-gal staining kit (G1580, Solarbio, Beijing, China). First, brain slices were rinsed twice with PBS and then fixed at room temperature for 15 min using β-galactosidase fixative. Then the brain slices were rinsed three times for 3 min each, and 1 mL of mixed staining solution was added, comprising 10 μL of β-galactosidase A, 10 μL of staining B, 930 μL of staining C, and 50 μL of X-Gal. Ambient conditions were maintained at 37 °C to facilitate overnight incubation. Finally, the staining solution was discarded, the film was sealed with sealing agent and photographed under a light microscope. The presence of small and uniform blue particles in the cytoplasm indicates β-galactosidase activity.

### Open field test (OFT)

The experimental setup for OFT consisted of a white, opaque cubic box measuring 45 cm × 45 cm × 45 cm, designed to minimize external visual cues and distractions. Each mouse was placed in the box with its back facing one of the walls to ensure consistent starting conditions across all animals. The mice were then allowed to explore the enclosure freely for 5 min. The total distance traveled, the time spent in the central region of the box, and the frequency of visits and distance covered within this area during exploration were recorded. The entire procedure was conducted under controlled lighting and noise conditions to eliminate any external influence on mouse behavior.

### Novel object recognition (NOR) test

The NOR apparatus is a square cube with sides measuring 45 cm, designed specifically for experiments and featuring non-transparent properties. The experimental procedure was divided into two phases: pre-experiment and post-experiment. On the first day, referred to as the acclimatization period, each mouse was placed in the setup, all facing the same sidewall direction, and was allowed to explore the apparatus freely for 5 min. Next day, the mice were put back to the arena at the same starting point and were given 5 min to explore object A and object B. Two hours after the familiarization period, the object B (familiar object) was replaced with object C (new object), and the mice were given another 5 min to explore both objects. After 24 h, the object C (now the familiar object) was replaced with object D (new object), and the mice were given again 5 min to explore both objects. The behavioral software VisuTrack (Shanghai Xinruan Technology Co., Ltd., China) was employed to accurately record mouse behaviors during exploration, including their interaction with both familiar and novel objects. Discrimination index was calculated as follows: discrimination index = Time Novel − Time Familiar/Time Novel + Time Familiar.

### Morris water maze (MWM)

The MWM is a widely recognized behavioral testing system produced by Komen Software Ltd. (Chengdu, China). This task, which relies on the functional integrity of the hippocampus, serves as a key tool for evaluating spatial memory capabilities. The experimental setup of MWM consisted of a circular tank measuring 1.2 m in diameter and 50 cm in height, filled with water of 23 ± 2 ℃. A square escape platform, measuring 10 cm × 10 cm with a height of 15 cm, was fixed in the tank with surface 1.5 cm below the water surface. Various posters were affixed to the wall of the tank. A video-tracking camera was positioned directly above the center of the pool to record mouse movements and trajectories. In the habituation period, each mouse was subjected to a training regimen that lasted for five consecutive days, four trials each day. In the training sessions, each mouse was positioned at various starting points within the pool, and was allowed 60 s to find the platform and stay there for 15 s. If the mice did not find the platform within 60 s, they were manually guided to the platform and stayed there for 15 s. The latency time, measured in seconds, for the mice to locate the hidden platform was documented following each trial during the learning sessions. Twenty-four hours after completion of the training session, the hidden platform was removed. In the probe trial, each mouse was introduced into the pool and observed for 60 s. The duration each mouse stayed in the target zone and the number of crossings of the platform site were recorded.

### Golgi staining

Golgi staining is a well-established technique for visualization of dendritic spines. Golgi staining was performed using the FD Rapid Golgi Staining Kit PK401 (FD NEURO TECHNOLOGIES, INC, Columbia, MO), according to the manufacturer’s instructions. Brain tissues were processed and stained, and hippocampal slices were imaged at varying magnifications (10 × , 20 × , and 40 ×) using an Olympus VS120 microscope.

### ELISA for Aβ40/42

Brain tissue was homogenized in cold PBS containing 5% BSA, 0.03% Tween-20, and protease inhibitor cocktail and centrifuged at 16,000 × *g* for 20 min at 4 °C. Aβ40/42 levels were determined using a mouse Aβ40/42 ELISA kit (Elabscience Biotechnology, Wuhan, China) according to the manufacturer’s instructions.

### Statistical analysis

Data are presented as mean ± SEM, derived from three or more independent experiments. Data were analyzed with the Student’s *t*-test for comparison between two groups, or with one-way ANOVA followed by LSD *post-hoc* test for multiple group comparison. Statistical analysis was conducted using the GraphPad Prism 8.0 software. *P* < 0.05 was considered as statistically significant.

## Results

### YAP1 is decreased and Nr4a1 is increased in the hippocampus of aged mice

**Fig. 1:**
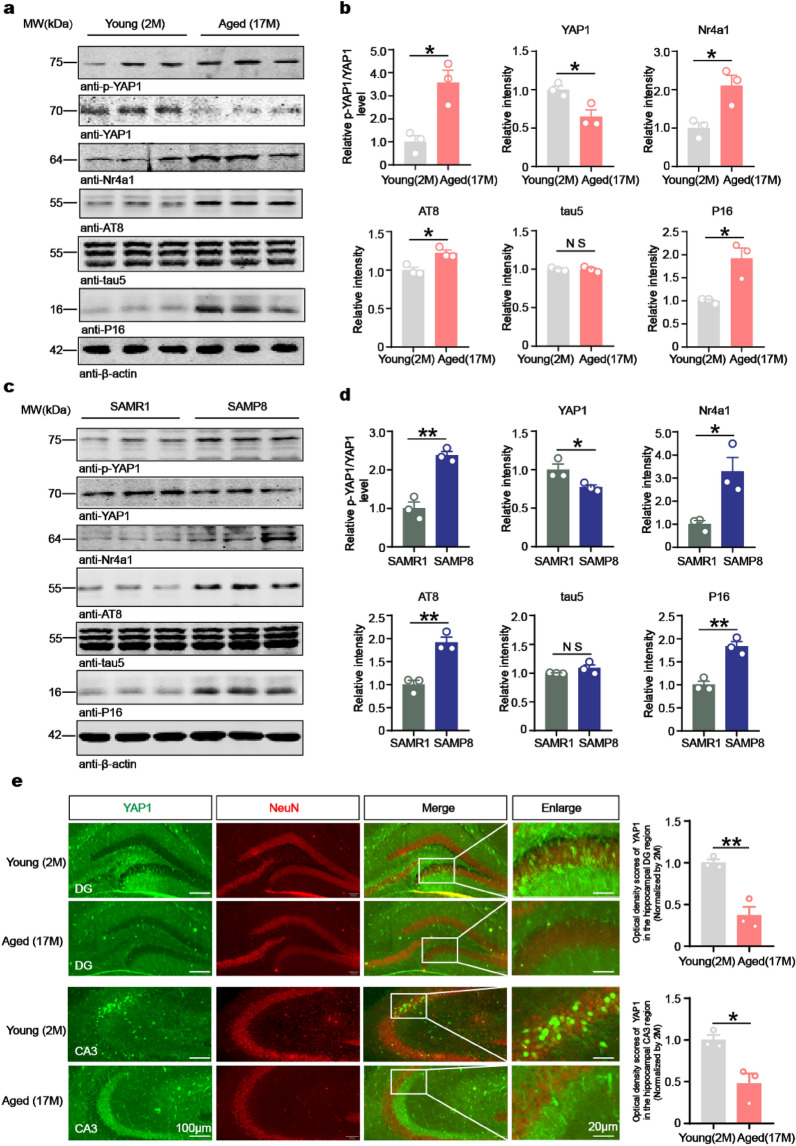
YAP1 was decreased and Nr4a1 was increased in aged mice. **a**–**d** Western blotting and quantification of the relative levels of YAP1, Nr4a1, tau5 and p16 normalized to β-actin, as well as p-YAP1/YAP1 and AT8/tau5 ratios, in the brains of 2-month-old and 17-month-old C57BL/6J mice (**a**, **b**) as well as SAMR1 and SAMP8 mice (**c**, **d**). **e** Immunofluorescent staining of NeuN (red) and YAP1 (green) in the hippocampal DG and CA3 regions of 2-month-old and 17-month-old C57BL/6J mice. Data are expressed as mean ± SEM, *n* = 3. *P < 0.05, **P < 0.01*,* NS, no significance

To monitor the changes of YAP1 level during aging, C57BL/6J mice of 2, 6, 9, 13, and 17 months of age were used for Western blotting. Results revealed that the YAP1 level began to decline at 13 months (Fig. S1a). In the hippocampus of 17-month-old mice, the p-YAP1 and p16 (a senescence marker [48, 49]) levels were significantly increased compared to the 2-month-old mice. However, the total YAP1 level was markedly decreased (Fig. 1a, b). These findings were further corroborated by immunofluorescence staining (Fig. S1b).

To identify the specific cell types in the hippocampus with reduced YAP1 and increased P16 expression, we performed immunofluorescence co-staining of YAP1 and p16, respectively, with neuronal (NeuN), microglial (IBA1), or astrocytic (GFAP) marker. We found that YAP1 was specifically reduced in neurons and p16 was significantly increased in the hippocampus of 17-month-old C57BL/6J mice. Notably, the co-localization of p16 was observed not only in neurons but also in microglia and astrocytes, indicating that p16 expression was elevated across multiple cell types within the hippocampus of 17-month-old C57BL/6J mice (Fig. S1c, d). We also observed some p16 staining with a linear pattern (Fig. S1d), which may correspond to the vasculature. As YAP1 directly regulates the transcription of *Nr4a1* [38], we next assessed the level of Nr4a1 in the hippocampi and observed elevated levels of both Nr4a1 and AT8 (pSer202/pThr205) in 17-month-old mice, consistent with prior results (Fig. 1a, b). In 17-month-old mice, phosphorylation of Hippo kinases, such as LATS1 and MST1, was significantly increased compared to younger counterparts (Fig. S1e, f), suggesting that YAP1 is downregulated and inactivated in the aged brain through a Hippo pathway-dependent mechanism. In cases of AD, neuronal YAP1 levels are notably diminished [50]. Indeed, immunofluorescence staining revealed that YAP1 expression was reduced in both the DG and the CA3 regions of 17-month-old mice (Fig. 1e).

To determine whether YAP1 is similarly downregulated and inactivated in age-related diseases such as AD, we analyzed YAP1 levels in the hippocampus of SAMP8 mice, a well-established transgenic model of AD by Western blotting [51, 52]. Results showed that both p-YAP1 and p16 levels were elevated, while the total YAP1 level was significantly reduced in AD mice (Fig. 1c, d). Immunofluorescence staining further confirmed increased p16 and decreased YAP1 levels in the CA1, CA3, and DG regions of the hippocampus in SAMP8 mice (Fig. S2b). Interestingly, YAP1 expression in the brains of SAMP8 mice was mainly found in dentate granule neurons (Fig. S2b), which is consistent with immunofluorescence staining of phosphorylated tau in SAMP8 (Fig. S2a), suggesting that YAP1 is probably associated with tau phosphorylation. Co-staining of YAP1 with NeuN, IBA1, and GFAP revealed that YAP1 was decreased mainly in neurons of SAMP8 mice (Fig. S2c). Consistent with these observations, phosphorylation of Hippo kinases LATS1 and MST1 was markedly increased in SAMP8 mice compared to wild-type controls (SAMR1) (Fig. S2d, e), further supporting the hypothesis that YAP1 downregulation occurs via the Hippo pathway. Collectively, these findings suggest that YAP1 was reduced in hippocampal lysates of both aged and AD model mice, potentially contributing to brain aging and the pathogenesis of AD.

### YAP1 negatively regulates the Nr4a1–AKT/GSK-3β axis and blocks senescence and tau hyperphosphorylation in primary neurons

**Fig. 2:**
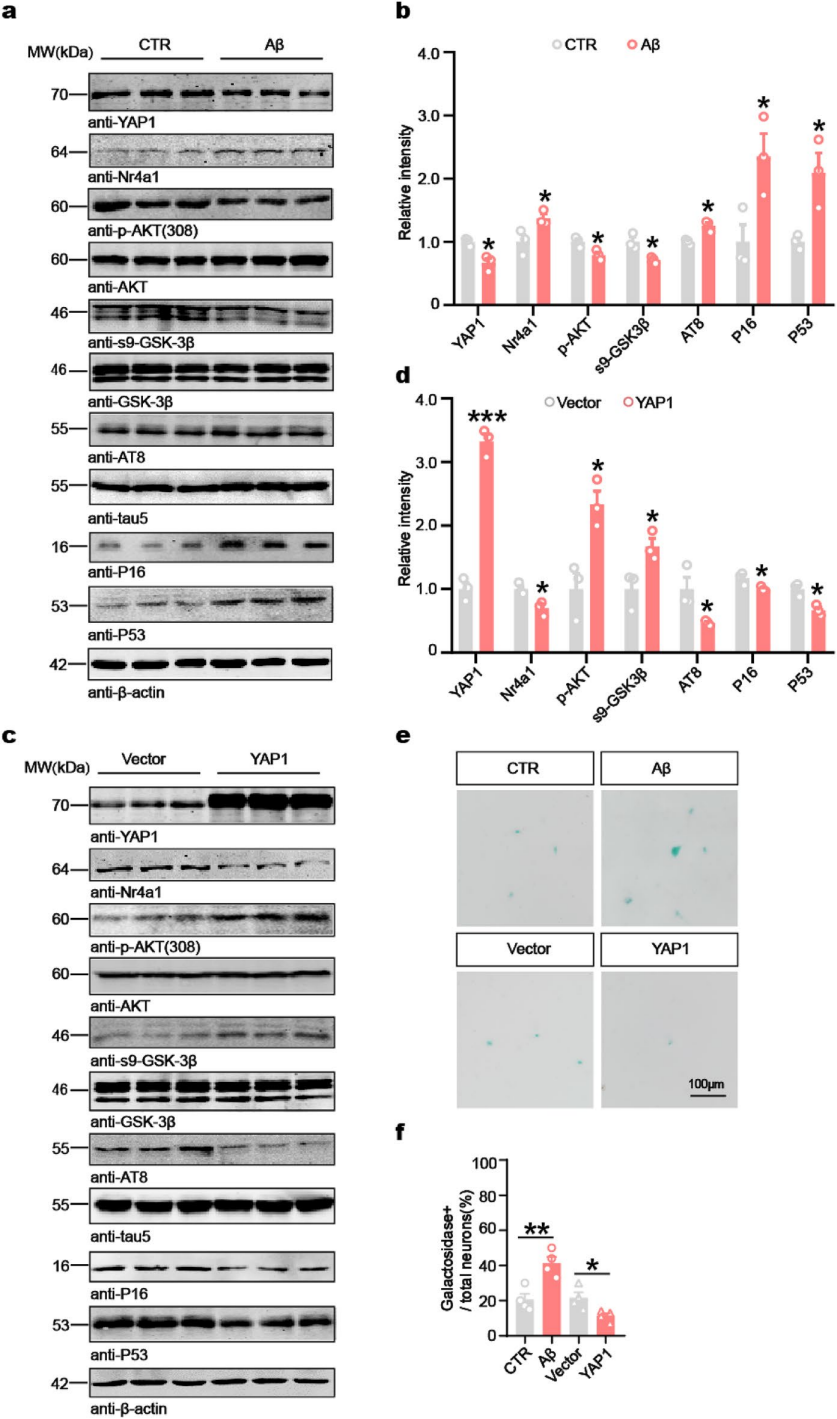
YAP1 negatively regulates the Nr4a1–AKT/GSK-3β axis and blocks senescence and tau hyperphosphorylation in primary neurons. **a**–**d** Western blotting and quantification of relative levels of YAP1, Nr4a1, p16 and p53 normalized to β-actin, as well as p-AKT/AKT, s9-GSK-3β/GSK-3β, and AT8/tau5 ratios in primary neurons with or without 5 μmol/L Aβ treatment (**a**, **b**), and in primary neurons with or without YAP1 overexpression (**c, d**). *n* = 3. **e, f** SA-β-gal staining of primary neurons with Aβ treatment or YAP1 overexpression, as well as control treatment (**e**) and quantification of the percentage of β-galactosidase-positive neurons over total neurons (**f**). *n* = 4. Data are expressed as mean ± SEM, *P < 0.05, **P < 0.01, ***P < 0.001

Previous research has indicated that YAP1 dysfunction, as a critical upstream regulator, may facilitate the progression of AD by modulating the expression of downstream genes [37]. To investigate YAP1 levels in senescent neurons, we established an Aβ-induced neuronal senescence model [11, 19] by treating primary neurons with 5 μmol/L Aβ for 24 h. The primary neurons exhibited clear signs of senescence, as confirmed by SA-β-gal staining (Fig. 2e, f). Consistent with in vivo findings, these senescent neurons displayed a significant reduction of YAP1 expression, accompanied by increased levels of Nr4a1, p16, and p53 (Fig. 2a, b). Downregulation of YAP1 in the early stages of AD is hypothesized to be a detrimental event, thus we examined the impact of YAP1 deletion and overexpression on key proteins involved in Aβ generation and tau phosphorylation. Results showed significantly increased tau phosphorylation at Ser202 and Thr205 in mice with Aβ-induced YAP1 reduction, whereas overexpression of YAP1 reversed this trend (Fig. 2c, d). Numerous studies have demonstrated that GSK-3β, a key kinase for tau protein phosphorylation, is activated by dephosphorylation at Ser9 and Ser389 or phosphorylation at Tyr216 [53–56]. GSK-3β is inactive when phosphorylated by AKT at Ser9 [57, 58]. Thus, we assessed the phosphorylation levels of AKT and GSK-3β using Western blotting analysis. Compared to the control group, Aβ treatment of hippocampal neurons resulted in markedly reduced phosphorylation of AKT at Thr308 and GSK-3β at Ser9 (Fig. 2a, b).

Overexpression of YAP1 in cultured primary neurons induced a decrease of β-galactosidase-positive neurons (Fig. 2e, f), and significantly increased phosphorylation of AKT at Thr308 and GSK-3β at Ser9 (Fig. 2c, d). These findings suggest that YAP1 deficiency promotes neuronal senescence in vitro, whereas its overexpression mitigates this process.

### Downregulation of YAP1 induces AD pathology and cognitive defects in C57BL/6J mice

**Fig. 3:**
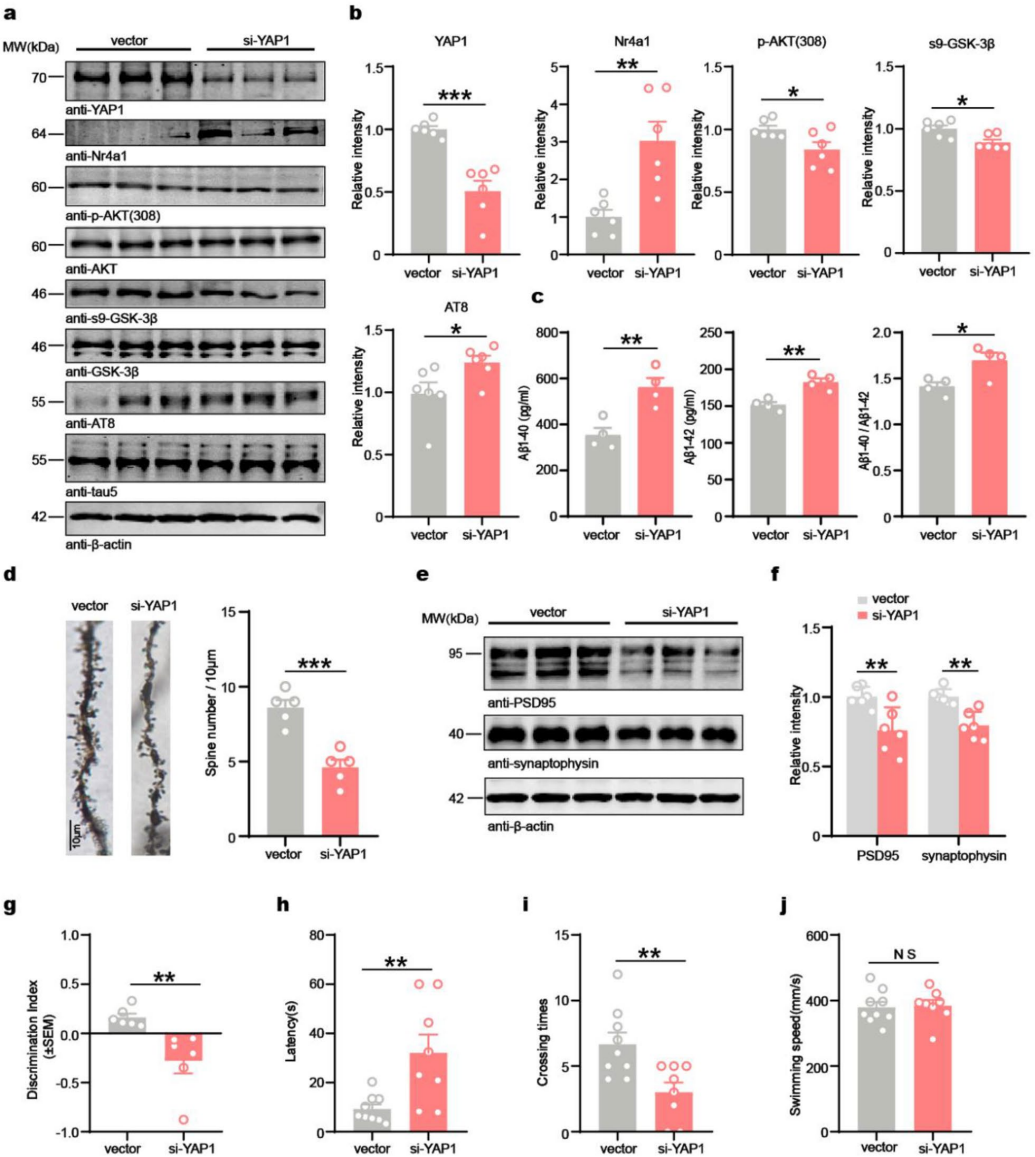
Downregulation of YAP1 induces AD pathologies and cognitive defects in C57BL/6J mice. **a**, **b** Western blotting and quantification of the relative levels of YAP1 and Nr4a1 normalized to β-actin, as well as p-AKT/AKT, s9-GSK-3β/GSK-3β, and AT8/tau5 ratios in the brains of vector and si-YAP1 group mice. *n* = 6. **c** Aβ1-40 and Aβ1-42 levels in the hippocampus determined by ELISA. *n* = 4. **d** Golgi-stained dendrites of CA3 neurons, and quantification of the average number of dendritic spines per neuron. *n* = 5 biologically independent samples. **e**, **f** Western blotting and quantification of the relative protein levels of PSD95 and synaptophysin in the hippocampus (*n* = 6). **g** NOR test in C57BL/6J mice that received AAV-si-YAP1 (*n* = 6).** h**, **i** Escape latency and number of platform crossings in the testing phase in MWM (*n* = 8–9).** j** Swimming speed throughout the MWM (*n* = 8–9). Data are expressed as mean ± SEM. *P < 0.05, **P < 0.01, ***P < 0.001, NS, no significance

To investigate whether decreased YAP1 levels would cause AD-like pathological damage and behavioral changes in vivo, we conducted bilateral AAV injections to selectively downregulate YAP1 within the CA3 region of the hippocampus in 2-month-old C57BL/6J mice. Behavioral tests and biochemical analyses were performed at 30 days after viral injection. Western blot analysis confirmed decreased YAP1 expression in the si-YAP1 group compared to the vector control group, indicating successful knockdown (Fig. 3a, b). The protein level of Nr4a1 was significantly elevated, while the phosphorylation of AKT and GSK-3β was decreased in the si-YAP1 group compared to the controls, leading to increased tau phosphorylation (Fig. 3a, b). ELISA assays revealed higher levels of Aβ1-40 and Aβ1-42 in the si-YAP1 group compared to the controls (Fig. 3c). Previous studies have demonstrated that YAP1 depletion induces cellular senescence in various tissues [34, 59]. Furthermore, both immunoblotting and immunofluorescence staining revealed that YAP1 knockdown led to increased expression of p16 (Fig. S3a, b).

Synaptic damage is an early indicator of AD progression and significantly impacts cognitive function [60, 61]. Golgi staining demonstrated reduced dendritic spine density in the si-YAP1 mice compared with the vector control group (Fig. 3d). Western blot indicated marked decreases in the levels of synaptic proteins PSD-95 and synaptophysin in the si-YAP1 mice compared to controls (Fig. 3e, f). The hallmark symptoms of AD include a gradual decline in cognitive abilities and memory function [62]. To assess the impact of YAP1 knockdown on learning and memory, we performed NOR test and MWM test to assess the hippocampus-dependent memory. Results of both NOR and MWM tests showed that YAP1 knockdown resulted in cognitive dysfunction (Fig. 3g–i), with no significant differences in locomotor activity between the two groups (Fig. 3j). The above data suggest that downregulation of YAP1 mimics AD-like cognitive deficits in wild-type mice and triggers early-onset cognitive decline.

### YAP1 overexpression ameliorates AD lesions in SAMP8 mice

**Fig. 4:**
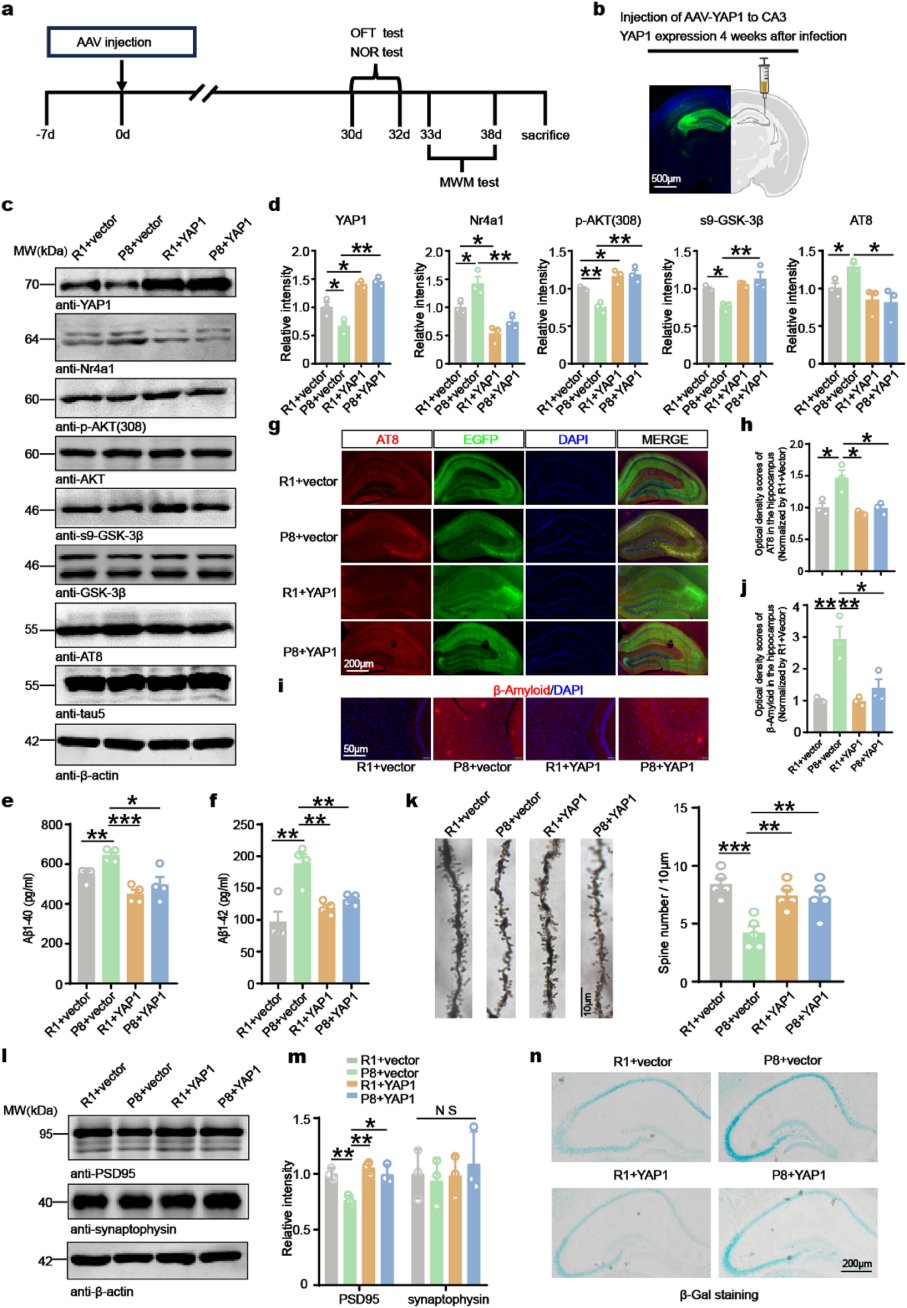
YAP1 overexpression ameliorates AD lesions in SAMP8 mice. **a** Time course of the experimental procedure.** b** Illustration of AAV-GFP or AAV-GFP-YAP1 injection into the CA3 region of SAMR1 or SAMP8 mice. GFP fluorescence in the hippocampus was observed one month after the injection (scale bar, 500 µm).** c, d** Western blotting and quantification of YAP1 and Nr4a1 levels normalized to β-actin, as well as p-AKT/AKT, s9-GSK-3β/GSK-3β, and AT8/tau5 ratios.* n* = 3 animals/group.** e, f** Aβ1-40 and Aβ1-42 levels in the hippocampus determined by ELISA (*n* = 4 animals/group).** g**–**j** Immunofluorescence staining for AT8 (red) and Aβ (red) in the hippocampus and frontal cortex and quantitative analysis of AT8- and Aβ-positive plaques (*n* = 3). **k** Golgi-Cox-stained dendrites of CA3 neurons and the average number of dendritic spines per neuron (*n* = 5 animals/group). **l**, **m** Western blotting and quantification of PSD95 and synaptophysin protein levels in the hippocampus (*n* = 3 animals/group). **n** SA-β-gal staining in the hippocampus. Data are expressed as mean ± SEM. *P < 0.05, **P < 0.01, ***P < 0.001*,* NS, no significance

To investigate the potential effects of elevating YAP1 expression in the hippocampi of the SAMP8 mouse model of AD, we stereotactically injected AAVs expressing full-length mouse YAP1 (AAV-YAP1) with the hSyn promoter into the hippocampi of 3-month-old male SAMP8 and SAMR1 mice (Fig. 4a). One month after virus injection, GFP fluorescence was specifically localized within the hippocampi, particularly in the CA3 region, confirming successful viral delivery (Fig. 4b). Western blot analysis showed efficient YAP1 expression, with increased levels in the AAV-YAP1 group compared to the AAV-vector group, accompanied by a decrease in Nr4a1 level. Overexpression of YAP1 restored AKT and GSK-3β phosphorylation, leading to a reduction in AT8 staining (Fig. 4c, d). Further, ELISA assays revealed that YAP1 overexpression resulted in reduced concentrations of Aβ peptides (Aβ40 and Aβ42) in SAMP8 mice (Fig. 4e, f).

Immunofluorescent staining showed stronger AT8 signals in the hippocampi of SAMP8 mice compared to control SAMR1 mice, further confirming tau hyperphosphorylation in SAMP8 mice. In contrast, no significant AT8 signal was detected in the YAP1 overexpression groups (Fig. 4g, h). A similar pattern was observed for Aβ aggregation, which was validated using an antibody specific to Aβ aggregates (Fig. 4i, j).

Golgi staining revealed a significant reduction in dendritic spine density in SAMP8 mice compared to controls. However, YAP1 overexpression attenuated this reduction (Fig. 4k). Furthermore, the decrease of postsynaptic protein PSD95 level observed in SAMP8 mice was partially reversed by YAP1 overexpression, while no significant changes were observed in presynaptic protein synaptophysin (Fig. 4l, m). These findings suggest that YAP1 overexpression prevents synaptic loss in SAMP8 mice by preserving dendritic spine density and maintaining levels of postsynaptic proteins.

It has been reported that overexpression of YAP1 attenuates alveolar epithelial cell senescence in vivo and in vitro [59]. To investigate the effects of YAP1 overexpression on neuronal aging, we assessed senescence-associated SA-β-gal levels in hippocampal neurons. We found a significant increase in SA-β-gal activity in SAMP8 mice compared to SAMR1 controls, indicating enhanced neuronal aging. However, overexpression of YAP1 in SAMP8 mice led to a reduction of SA-β-gal level (Fig. 4n), suggesting that YAP1 loss accelerates neuronal aging. Consistent with these findings, immunoblotting analysis for p16 also showed similar trends (Fig. S4a). To further investigate the cellular localization of p16, we performed immunofluorescence co-staining for p16, NeuN, IBA1, and GFAP. The assay revealed significant co-localization of p16 with neurons and glial cells in SAMP8 mice. However, YAP1 overexpression reduced the co-localization of p16 with these cell types (Fig. S4b–d).

**Fig. 5:**
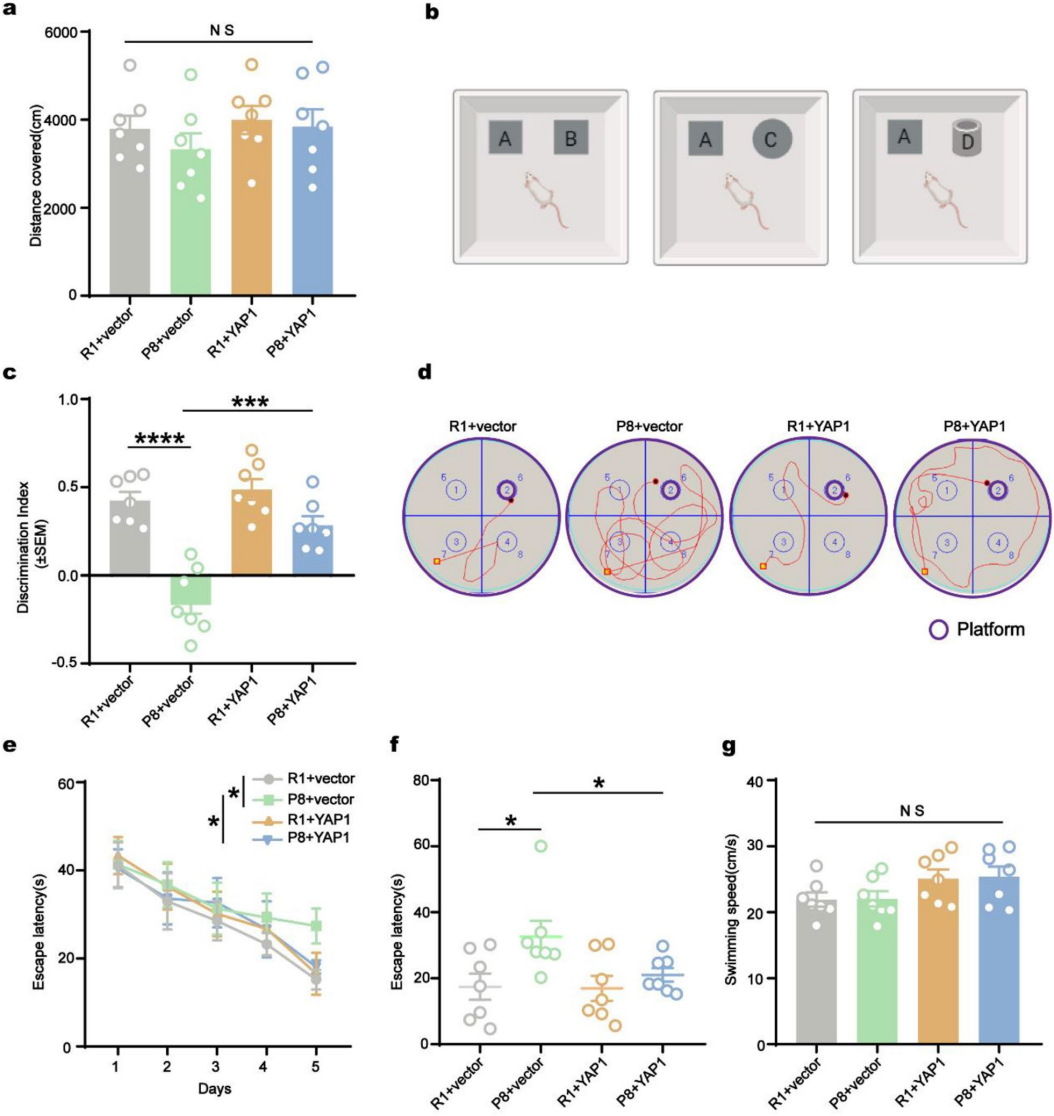
Overexpression of YAP1 improves cognitive function of SAMP8 mice. **a** The total distance traveled in the open-field test. **b** Illustration of experimental training in the new object recognition test. **c** Recognition index for the new object recorded over a 24 h period in the NOR test. **d** Swimming path to locate the platform for the first time in the testing phase. **e, f** The escape latencies in the MWM test during training from day 1 to day 5 (**e**) and during testing on day 6 (**f**). **g** Swimming speed on day 6 in the MWM test. Data are expressed as mean ± SEM, *n* = 7, *P < 0.05, ***P < 0.001*,* ****P < 0.0001*,* NS, no significance

To evaluate the potential benefits of exogenous YAP1 in alleviating AD symptoms, we conducted a series of behavioral tests one month after AAV injection (Fig. 4a). OFT results revealed no significant changes in motor activity among groups (Fig. 5a). Subsequently, we evaluated hippocampal cognitive function using NOR (Fig. 5b). Mice of the SAMP8 + vector group exhibited a notable decrease in recognition index, whereas AAV-YAP1 treatment significantly improved the ability of SAMP8 mice to recognize new targets (Fig. 5c). Moreover, MWM test demonstrated that YAP1 overexpression effectively ameliorated learning and memory impairments in SAMP8 mice. Specifically, on day 5 of training phase, the escape latency was significantly reduced (Fig. 5e), indicating enhanced learning. In the testing phase on day 6, the latency to reach the platform was considerably shortened (Fig. 5d, f), while the swimming speed did not differ significantly between groups, ruling out potential dyskinesia as a confounding factor (Fig. 5g). These results suggest that upregulation of YAP1 expression enhances cognitive function in an AD mouse model.

### Downregulation of Nr4a1 attenuates tau phosphorylation and cognitive impairments in SAMP8 mice

**Fig. 6:**
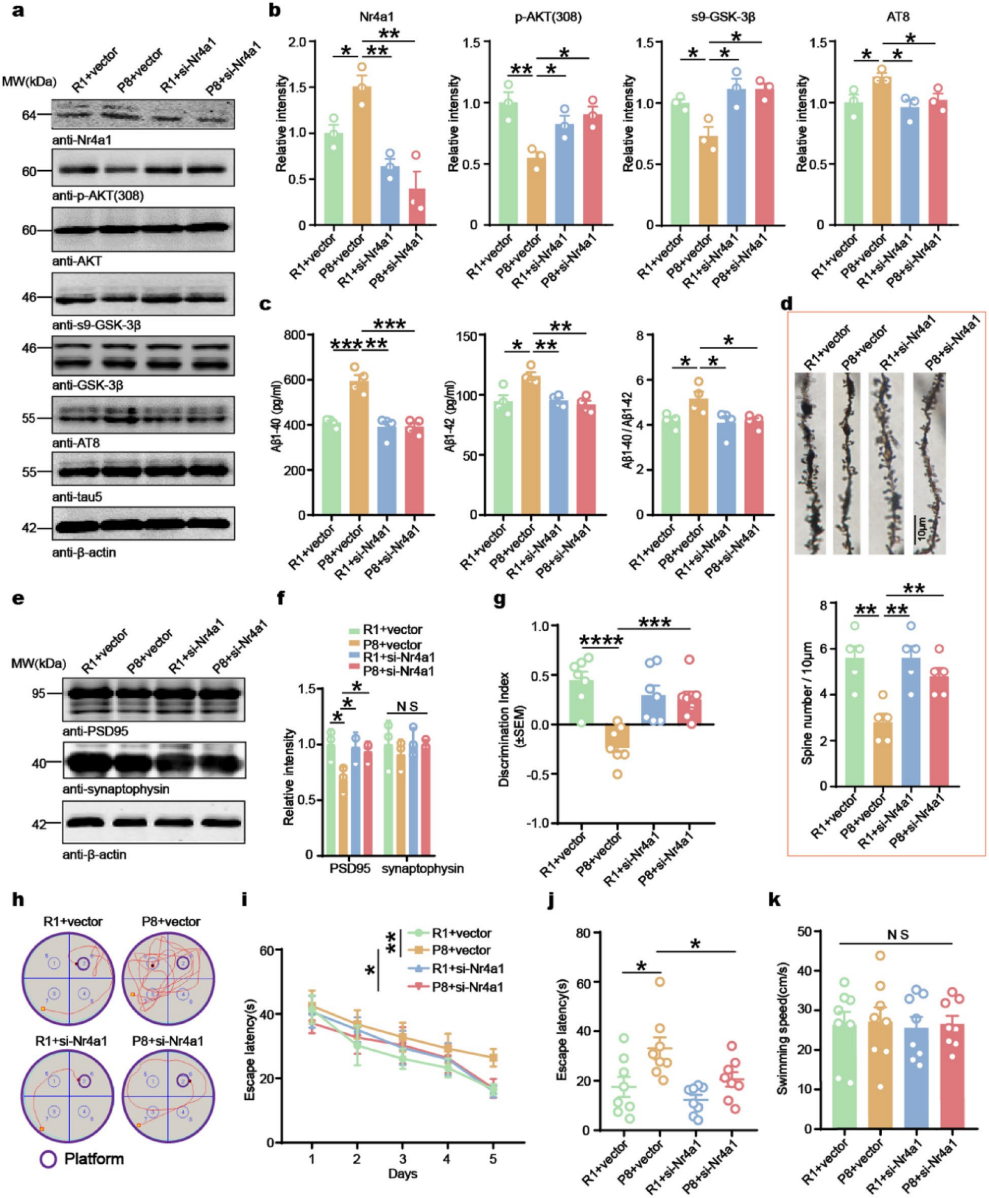
Downregulation of Nr4a1 attenuates tau phosphorylation and cognitive impairments in SAMP8 mice. **a**, **b** Western blotting and quantification of Nr4a1 level normalized to β-actin, p-AKT/AKT, s9-GSK-3β/GSK-3β, and AT8/tau5 ratios (*n* = 3 animals/group). **c** Aβ1-40 and Aβ1-42 levels in the hippocampus determined by ELISA (*n* = 4 animals/group). **d** Golgi-Cox-stained dendrites of CA3 neurons in both groups and quantification of the average number of dendritic spines per neuron. *n* = 5 biologically independent samples. **e**, **f** Western blotting and quantification of the relative protein levels of PSD95 and synaptophysin in the hippocampus (*n* = 3 animals/group). **g** Recognition index for the new object recorded over a 24 h period in the NOR test (*n* = 7 animals/group). **h** Representative swimming paths to locate the platform for the first time during the testing phase. **i**, **j** The escape latencies in the MWM test during training from day 1 to day 5 (**i**) and during testing on day 6 (*n* = 8 animals/group) (**j**). **k** Swimming speed on day 6 in the MWM test (*n* = 8 animals/group). Data are expressed as mean ± SEM. *P < 0.05, **P < 0.01, ***P < 0.001, ****P < 0.0001. NS, no significance

It has been reported that YAP1 directly exerts a negative regulatory effect on the expression of Nr4a1 [38]. To investigate whether Nr4a1 mediates the reduction of AD-like lesions caused by YAP1 overexpression, Nr4a1 was knocked down in 3-month-old SAMR1/SAMP8 mice by injecting AAV-si-Nr4a1 or AAV vector bilaterally in the hippocampi of mice. Cognitive function was assessed one month post-injection. Western blot analysis revealed a significant decrease of Nr4a1 level in the hippocampi of mice injected with AAV-si-Nr4a1 compared to those injected with AAV-vector, confirming successful viral infection. The SAMP8 mice exhibited reduced phosphorylation of AKT and GSK-3β as well as increased tau phosphorylation compared to the SAMR1 mice, which were rescued upon knockdown of Nr4a1 (Fig. 6a, b). ELISA quantification further revealed elevated levels of Aβ1-40 and Aβ1-42 in the SAMP8 group compared to SAMR1 mice, which were significantly attenuated following Nr4a1 knockdown (Fig. 6c).

To examine the impact of Nr4a1 on synaptic proteins, we performed Golgi staining on hippocampal neurons. The SAMP8 mice exhibited significantly reduced dendritic complexity compared to SAMR1 controls, which was partially restored upon Nr4a1 knockdown (Fig. 6d). Furthermore, the decline in PSD95 protein level observed in SAMP8 mice was mitigated by Nr4a1 knockdown, as confirmed by Western blotting (Fig. 6e, f).

Consistent with extensive synaptic degeneration, SAMP8 mice exhibited a significantly lower cognitive recognition index in the NOR test and increased escape latency in the MWM. However, these impairments were reversed by Nr4a1 knockdown (Fig. 6g–j). Notably, the swimming speed remained unaffected across all experimental groups (Fig. 6k). Collectively, these findings provide strong support for the hypothesis that the Nr4a1-mediated dendritic branching and spine development defects play a crucial role in YAP1-induced AD-like lesions.

### Genetic and chemical inhibition of Nr4a1 blocked the YAP1 deficiency-induced AD pathologies and cognitive impairment in C57BL/6J mice

**Fig. 7:**
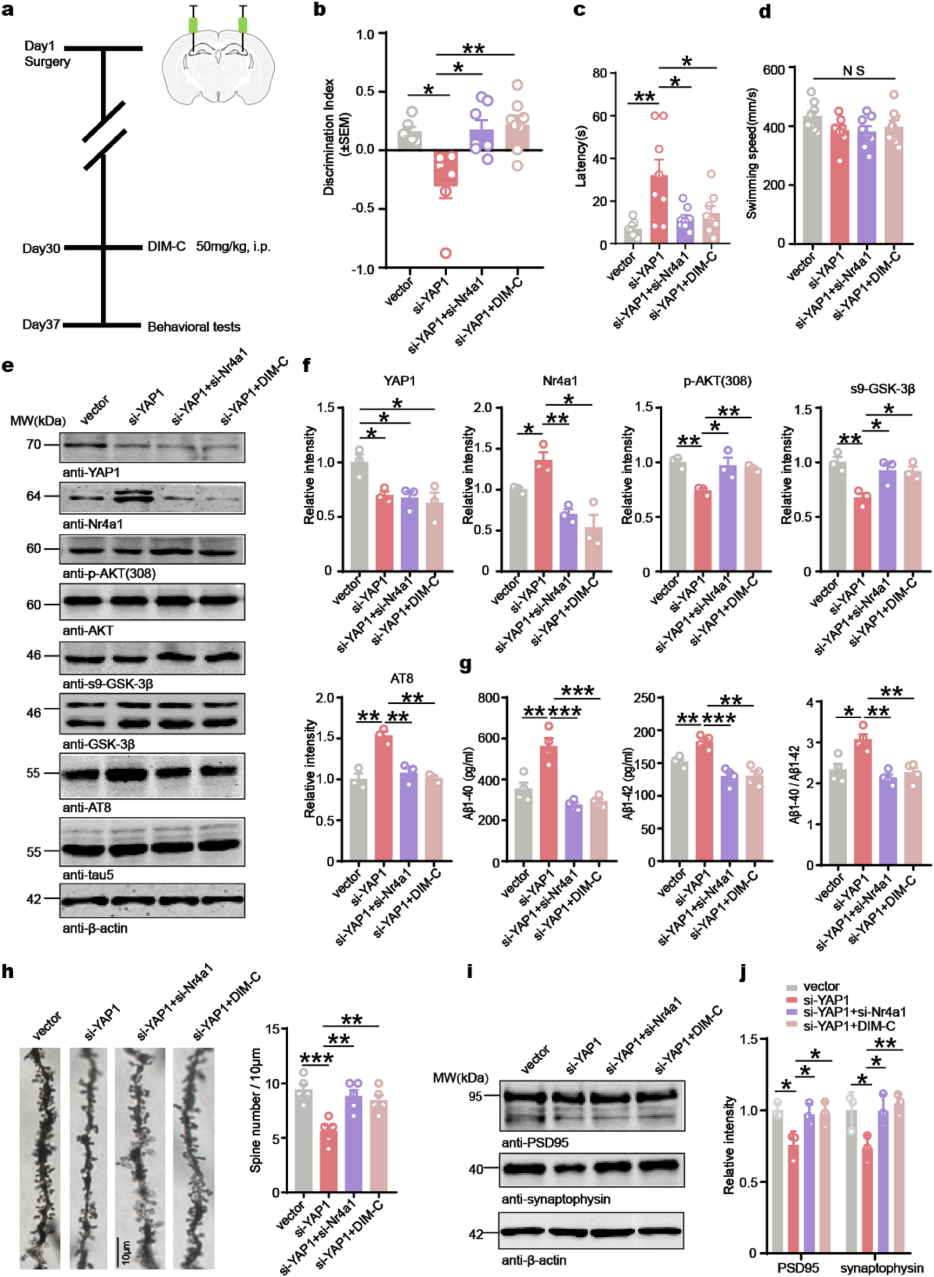
Genetic and chemical inhibition of Nr4a1 blocks the YAP1 deficiency-induced AD pathologies and cognitive impairment in C57BL/6J mice. **a** Time course of the experimental procedure. **b** The recognition index for the new object recorded over a 24 h period in the NOR test (*n* = 6–8 animals/group). **c** The latency to first cross the target platform on day 6 of MWM (*n* = 8 animals/group). **d** Swimming speed on day 6 of MWM (*n* = 8 animals/group).** e, f** Western blotting and quantification of YAP1 and Nr4a1 levels normalized to β-actin, as well as p-AKT/AKT, s9-GSK-3β/GSK-3β and AT8/tau5 ratios (*n* = 3 animals/group). **g** Aβ1-40 and Aβ1-42 levels in the hippocampus determined by ELISA (*n* = 4 animals/group). **h** Golgi staining and quantitation of spine number in the dentate gyrus of the hippocampus (*n* = 5 animals/group).** i, j** Western blotting and quantification of PSD95 and synaptophysin protein levels in the hippocampus (*n* = 3 animals/group). Data are expressed as mean ± SEM. *P < 0.05, **P < 0.01, ***P < 0.001. NS, no significance

To further confirm that YAP1 knockdown induces AD-like lesions and cognitive dysfunction through upregulation of Nr4a1 expression, we investigated whether reducing Nr4a1 levels could rescue the AD-like lesions associated with YAP1 deficiency in vivo. For this purpose, we bilaterally injected AAV-siYAP1 or AAV-siYAP1 + AAV-si-Nr4a1 into the hippocampal CA3 region of 2-month-old C57BL/6J mice. After 30 days, the AAV-siYAP1 group was treated with DIM-C (50 mg/kg, i.p.) and all animals were subjected to a series of behavioral tests (Fig. 7a). In the OFT, no significant differences in the total movement distance were observed across the four experimental groups (Fig. S5a). However, in the NOR test, the si-YAP1 group exhibited a significant decline in the cognitive discrimination index compared to the vector group, indicating impaired cognitive function. Notably, both Nr4a1 knockdown and DIM-C treatment substantially increased the cognitive discrimination index compared to the AAV-siYAP1 group (Fig. 7b). During the MWM test, on day 5 of training, the si-YAP1 mice displayed impairment of learning ability, as evidenced by significantly prolonged latency to locate the hidden platform (Fig. S5b). However, Nr4a1 knockdown or inhibition significantly reduced this latency (Fig. 7c), with no significant differences in the swimming speed across the four groups (Fig. 7d). These findings suggest that Nr4a1 inhibition may have therapeutic potential for mitigating YAP1-induced cognitive impairments in mice.

Subsequent analysis confirmed effective YAP1 knockdown by AAV-siYAP1. Western blotting also revealed an increase in Nr4a1 expression following YAP1 knockdown, while Nr4a1 levels were reduced upon either Nr4a1 knockdown or DIM-C treatment. Additionally, Western blot analysis demonstrated a significant reduction in the phosphorylation of AKT and GSK-3β in the si-YAP1 group compared to the vector control, accompanied by increased tau phosphorylation at Ser202 and Thr205. Knockdown or inhibition of Nr4a1 restored the phosphorylation of AKT and GSK-3β, leading to a reduction in hyperphosphorylated tau protein level (Fig. 7e, f). Additionally, we observed a significant increase in p16 protein level in the si-YAP1 group, and knockdown or inhibition of Nr4a1 partially reduced p16 protein level (Fig. S5c, d).

ELISA assays revealed elevated levels of Aβ1-40 and Aβ1-42 in the si-YAP1 group compared to controls, which were partially reduced by either Nr4a1 knockdown or DIM-C treatment (Fig. 7g). Golgi staining demonstrated a significant loss of dendritic spines in the si-YAP1 group, which was effectively mitigated by si-Nr4a1 knockdown or DIM-C treatment (Fig. 7h). Consistent with these findings, levels of synapse-related proteins, including PSD95 and synaptophysin, were decreased in the si-YAP1 group; however, Nr4a1 inhibition partially reversed these reductions (Fig. 7i, j). Together, these results suggest that inhibition of Nr4a1 prevents synaptic damage induced by si-YAP1 in mice by preserving dendritic spine density and maintaining the expression of postsynaptic proteins.

## Discussion

Previous studies have established that downregulation of YAP1 leads to premature senescence in various cell types [30, 34, 36]. Our findings corroborate these observations, demonstrating that YAP1 is downregulated and inactivated in aging neurons, both in vivo and in vitro, mediated by the Hippo signaling pathway. Moreover, while earlier research suggested that YAP1 is sequestered within cytoplasmic amyloid aggregates associated with AD pathology [50], our research confirmed that YAP1 is predominantly expressed in neurons, consistent with previous reports [63–67].

Although senescent cells exacerbate the pathological processes associated with neurodegenerative diseases [68], the precise role of aging neurons in both the aging process and AD remains unclear. For instance, clearance of senescent retinal cells using dasatinib preserves retinal structure and function following elevated intraocular pressure [69]. Accumulation of senescent neurons contributes to tau protein aggregation and promotes the progression of neurodegenerative diseases [18], while Aβ induces neuronal senescence, creating a mutually reinforcing cycle [70, 71]. In 5 × FAD mice, Aβ exacerbates both neuronal senescence and cognitive impairment [17]. As organisms age, senescent cells release senescence-associated secretory phenotype factors, which trigger inflammation and foster an environment conducive to disease development [72]. Notably, targeting these senescent cells holds promise for extending the healthspan [73]. In the current study, YAP1 levels in neurons were significantly reduced in both aged mice and AD models due to the activation of Hippo kinase. In our study, YAP1 expression was specifically reduced in neurons, while p16 expression was upregulated in neurons, astrocytes, and microglia. Consistent with previous findings, these results suggest that senescence in astrocytes and microglia may be regulated by mechanisms independent of YAP1 signaling [74–77]. Furthermore, deletion of YAP1 in neurons accelerated brain aging, whereas activation of YAP1 mitigated this process. These findings suggest that reduced YAP1 expression in aging neurons may be a critical factor in the pathogenesis of brain aging and AD progression.

In YAP1-deficient mice, we observed cognitive and memory impairments resembling those seen in AD, accompanied by the loss of postsynaptic spine structures, tau phosphorylation, and increased Aβ production. The integrity of these postsynaptic spines is critical for synaptic function and is closely associated with cognitive deficits in AD [78]. The nuclear receptor gene *Nr4a1* plays a key role in regulating dendritic spine density in CA1 neurons, with its overexpression leading to a significant reduction of spine density [79]. Previous studies have demonstrated that the YAP/TAZ–TEAD complex directly inhibits Nr4a1 transcription [38]. PI3K and AKT are recognized as downstream effectors of Nr4a1. In the present study, we found that GSK-3β serves as a downstream target within the PI3K/AKT signaling pathway, where PI3K activation leads to phosphorylation of AKT at specific residues, thereby activating it. In turn, AKT phosphorylates GSK-3β at Ser9, inhibiting its activity and preventing tau hyperphosphorylation [57]. Disruption of this pathway has been linked to excessive tau phosphorylation in AD models [58, 80].

Here, Western blot analysis revealed a reduction in the phosphorylation of PI3K and AKT, suggesting that dysfunction of the PI3K/AKT/GSK-3β pathway may contribute to tau hyperphosphorylation in YAP1-deficient mice. This hypothesis warrants further investigation. Additionally, we detected elevated levels of Aβ1-40 and Aβ1-42, which align with findings observed in AD models. Excessive GSK-3β activity facilitates BACE1 expression and promotes nuclear translocation of NF-κB/p65, thereby enhancing Aβ production and accumulation, as well as the amyloidogenic processing of APP [81, 82]. In renal diseases, particularly with aging, GSK-3β expression and activity are significantly upregulated in podocytes, and this aberrant activity is closely associated with the aging process. Studies indicate that targeting and inhibiting GSK-3β could effectively mitigate senescence in these cells [83, 84]. Furthermore, these findings imply that GSK-3β activation may play a critical role in neuronal aging in YAP1-deficient mice.

Various strategies have been developed to selectively eliminate senescent cells and ameliorate age-related conditions, including senolytic therapies such as dasatinib and quercetin [4, 11]. Our research indicates that inhibition of YAP1 in mice leads to accelerated neuronal senescence and cognitive impairment. Conversely, blocking Nr4a1 with DIM-C in AD mouse models and aged mice results in partial cognitive improvement. This evidence suggests that activation of the YAP1–Nr4a1 pathway may slow brain aging and mitigate neurodegenerative diseases associated with aging. However, several studies indicate that both YAP1 and Nr4a1 are implicated in tumorigenesis [38, 85, 86]. Overactivation of the YAP1–Nr4a1 pathway could promote tumor growth, whereas moderate activation may facilitate the restoration of aging neurons. Therefore, careful modulation of YAP1 is essential for managing brain aging and neurodegenerative diseases. Further research is necessary to refine these therapeutic approaches.

## Conclusions

Our study demonstrated that in aging neurons, the YAP1 signaling pathway is significantly diminished and inactivated due to activation of the Hippo kinase. YAP1 deficiency contributes to neuronal senescence. In both aged mice and AD model mice, overexpression of YAP1 mitigates the progression of neuronal aging. This intervention not only delays the onset of neuronal senescence, but also leads to significant improvements in cognitive function. By elevating YAP1 levels, we can effectively counteract both the aging process and cognitive decline associated with AD. Our findings reveal previously unrecognized mechanisms for addressing neuronal aging. By elucidating this novel pathway, our findings open new avenues for identifying potential therapeutic targets aimed at alleviating brain aging. Furthermore, these results present promising prospects for combating various neurodegenerative disorders.

## Supplementary Information

**Additional file 1**. **Figure S1**. The Hippo signaling pathway was activated in the hippocampal neurons of aged C57BL/6J mice. **Figure S2**. The Hippo signaling pathway was activated in the hippocampal neurons of SAMP8 mice. **Figure S3**. Knockdown of YAP1 in C57BL/6J mice resulted in increased p16 in the hippocampus. **Figure S4**. overexpression of YAP1 led to a reduction of p16 in the hippocampus of SAMP8 mice. **Figure S5**. Nr4a1 was required for YAP1 deficiency to induce cognitive impairment and neuronal senescence.

## Data Availability

The datasets used and/or analyzed during the present study are available from the corresponding author upon reasonable request.

## Abbreviations

LOAD: Late-onset Alzheimer’s disease
AD: Alzheimer’s disease
YAP1: Yes-associated protein 1
Nr4a1: Nuclear receptor subfamily 4 group A member 1
SAMP8: Senescence-accelerated mouse prone-8
SAMR1: Senescence-accelerated-resistant mouse
Aβ: Beta-amyloid
NFTs: Neurofibrillary tangles
GSK‑3β: Glycogen synthase kinase‑3β
PI3K: Phosphatidylinositol-3 kinase
AKT: Protein kinase B
MWM: Morris water maze
NOR: Novel object recognition
OFT: Open field test
DIM-C: DIM-C-pPhOH
TEAD: Transcriptional enhanced associate domain
MST1: Mammalian sterile 20 (STE20)-like kinase 1
LATS1: Large tumor suppressor homolog 1
WT: Wild-type
