# Supplementary material for: Aging-dependent YAP1 reduction contributes to AD pathology by upregulating the Nr4a1-AKT/GSK-3β axis
Source: Transl Neurodegener. 2025 Jun 4;14:29. doi: 10.1186/s40035-025-00487-4 (PMC12135490; doi:10.1186/s40035-025-00487-4)
Supplement: Supplementary file 1 — Additional file 1. Figure S1. The Hippo signaling pathway was activated in the hippocampal neurons of aged C57BL/6J mice. Figure S2. The Hippo signaling pathway was activated in the hippocampal neurons of SAMP8 mice. Figure S3. Knockdown of YAP1 in C57BL/6J mice resulted in increased p16 in the hippocampus. Figure S4. overexpression of YAP1 led to a reduction of p16 in the hippocampus of SAMP8 mice. Figure S5. Nr4a1 was required for YAP1 deficiency to induce cognitive impairment and neuronal senescence. [file 40035_2025_487_MOESM1_ESM.docx]

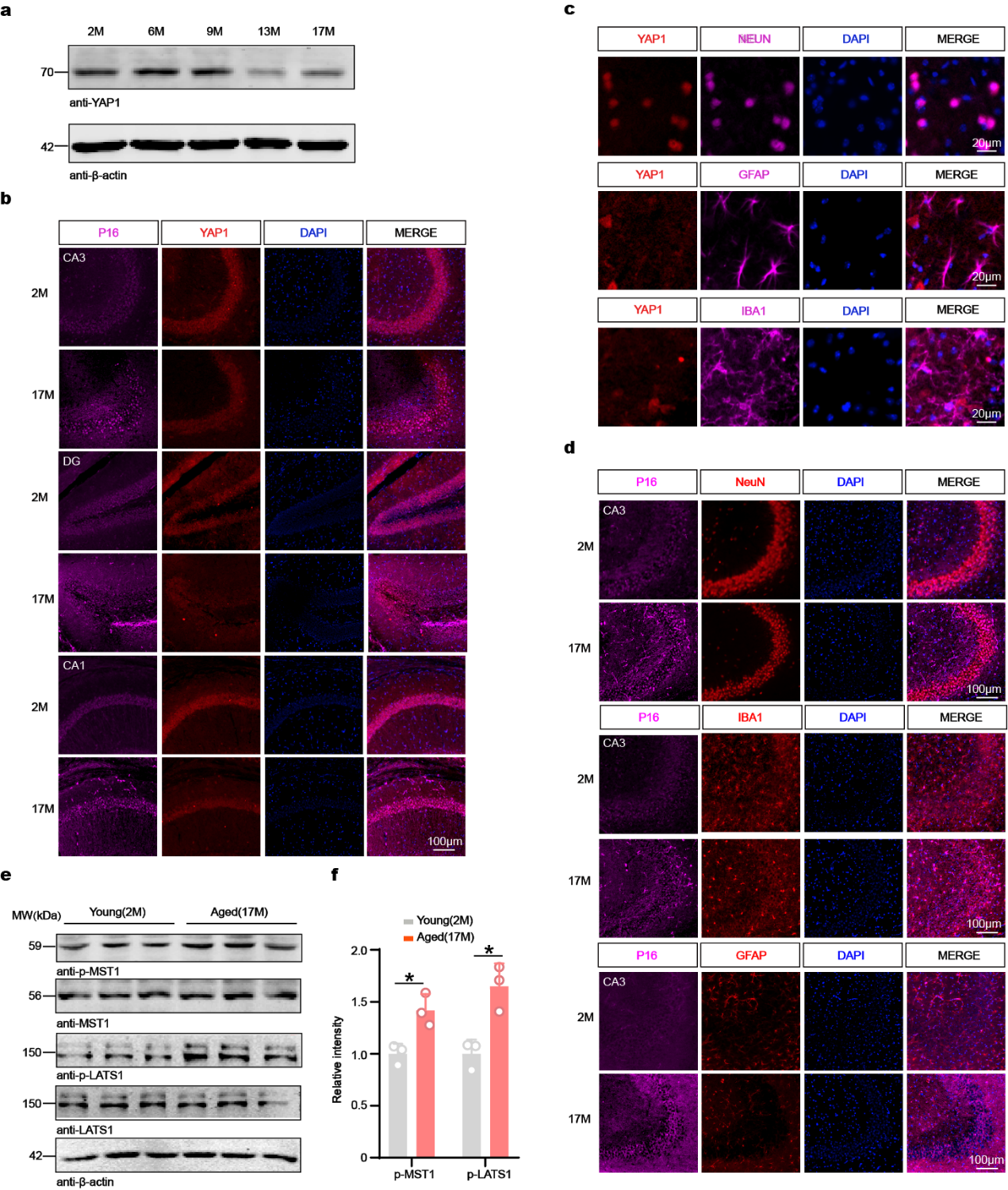


**Figure S1. The Hippo signaling pathway was activated in the hippocampal neurons of aged C57BL/6J mice**

**a**Western blot of YAP1 expression levels in C57BL/6J mice at ages 2, 6, 9, 13, and 17 months. **b** Co-localization of p16 and YAP1 in the CA3,DG,CA1 region of the hippocampus in 2-month-old and 17-month-old C57BL/6J mice, (scale bar=100 µm).**c** Immunofluorescence analysis of YAP1 co-localization with NeuN, IBA1, and GFAP in the hippocampus of 2-month-old C57BL/6J mice, (scale bar=20 µm).**d** Immunofluorescence staining showing co-localization of p16 with NeuN, IBA1, and GFAP in 2-month-old and 17-month-old C57BL/6J mice, (scale bar=100 µm). **e-f** Western blot and quantification of p-MST1, MST1, p-LATS1, and LATS1 expression in the hippocampus of 2-month-old and 17-month-old C57BL/6J mice(*n* = 3 per group, normalized to 2M mice); p-MST1 was normalized to MST1 and p-LATS1 was normalized to LATS1. Data were mean ± SEM. **p <* 0.05.


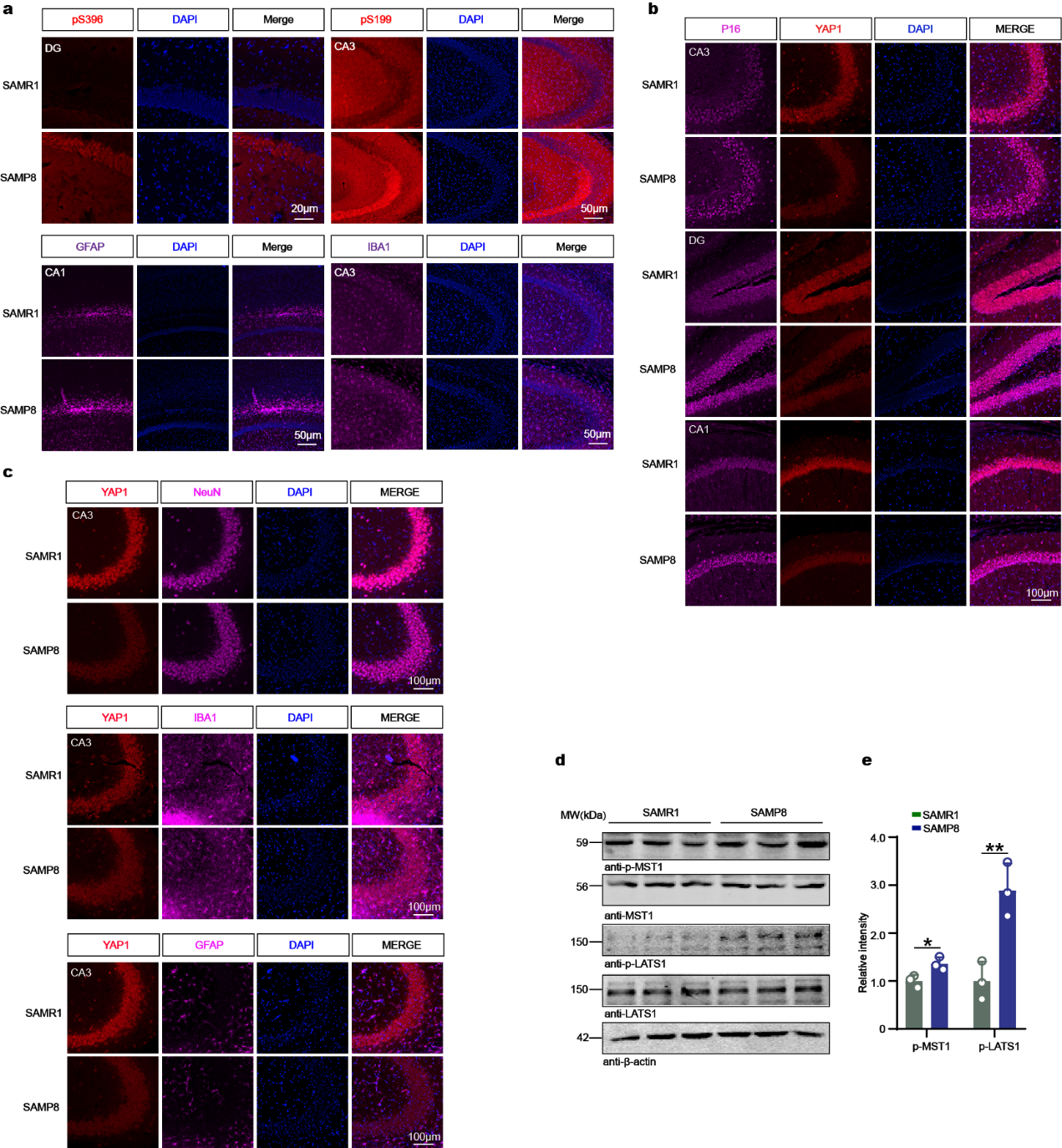


**Figure S2. The Hippo signaling pathway was activated in the hippocampal neurons of SAMP8 mice**

**a** Immunofluorescence analysis of pS396,pS199,GFAP and IBA1 in SAMP8 mice, (scale bar=20 µm, 50 µm)). **b** Co-localization of p16 and YAP1 in the CA3, DG, CA1 regions of the hippocampus in SAMP8 mice, (scale bar=100 µm).**c** Immunofluorescence analysis of YAP1 co-localization with NeuN, IBA1, and GFAP in SAMR1 and SAMP8 mice, (scale bar=100 µm). **d-e** Western blot and quantification of p-MST1, MST1, p-LATS1, and LATS1 expression in the hippocampus of SAMR1 and SAMP8 mice, (*n*= 3 per group, normalized to SAMR1 mice); p-MST1 was normalized to MST1 and p-LATS1 was normalized to LATS1. Data were mean ± SEM. **p <* 0.05, ***p <*0.01.


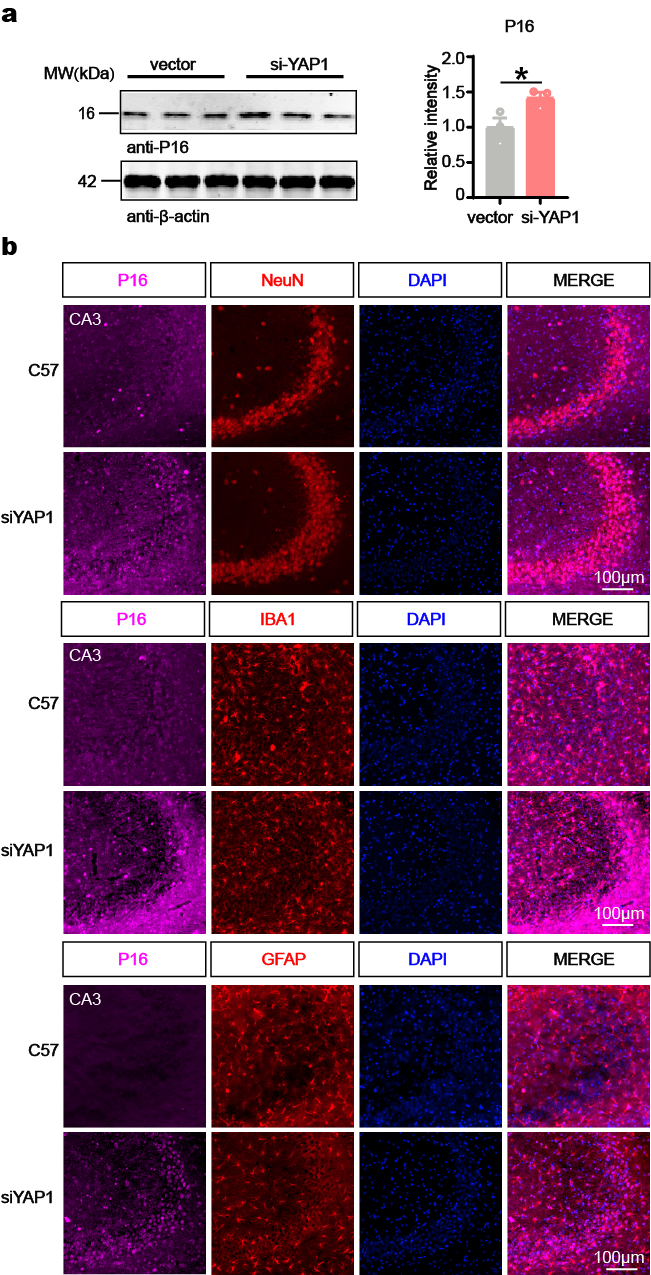


**Figure S3. Knockdown of YAP1 in C57BL/6J mice resulted in increased p16 in the hippocampus**

**a** Western blot and quantification of p16 expression in the hippocampus of the vector and si-YAP1 groups, (*n*= 3 per group).**b** Immunofluorescence analysis of p16 co-localization with NeuN, IBA1and GFAP in the vector and si-YAP1 groups mice, (scale bar=100 µm). Data are expressed as the mean ± SEM. **p* < 0.05.


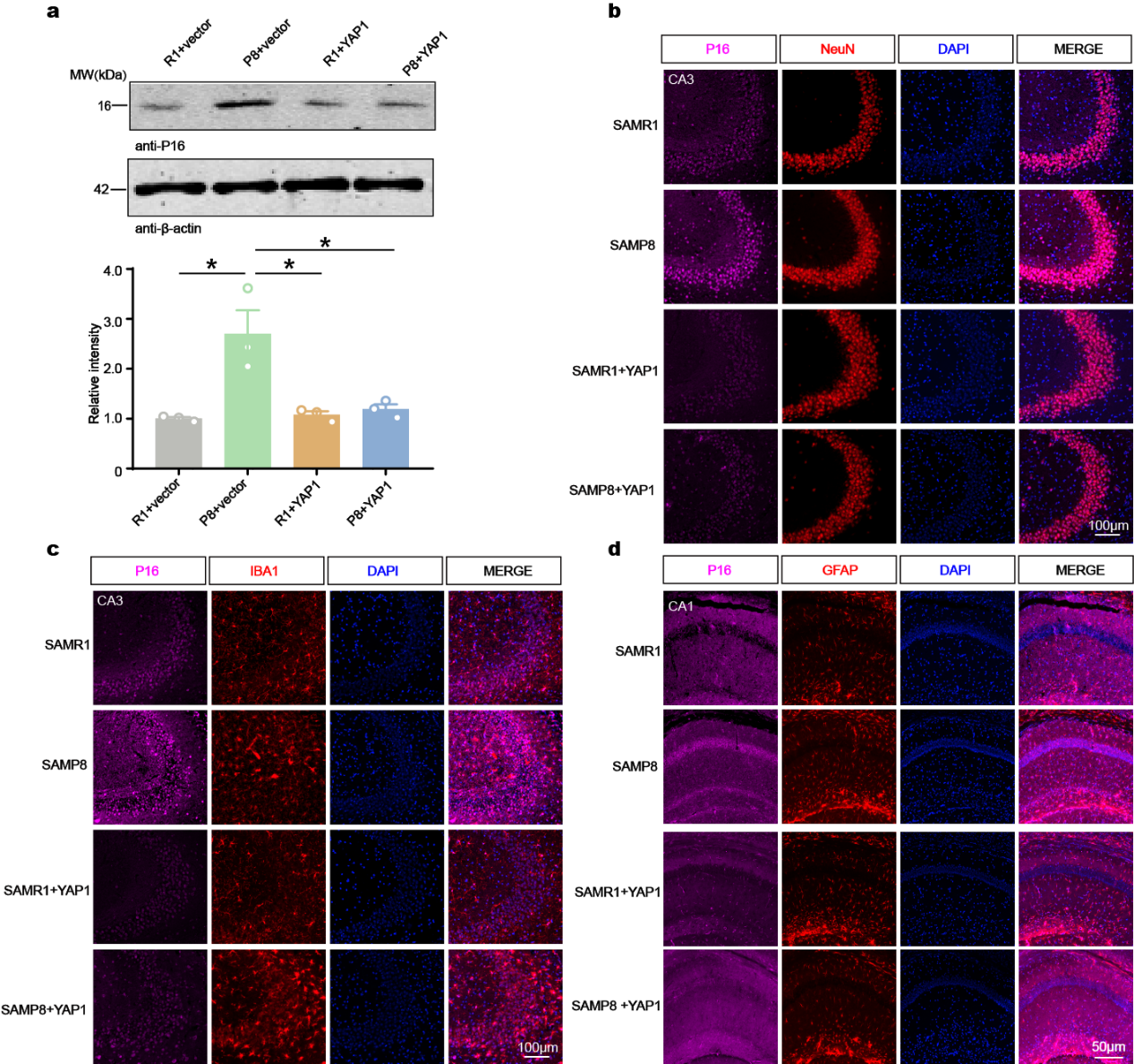


**Figure S4. overexpression of YAP1 led to a reduction of p16 in the hippocampus of SAMP8 mice**

**a** Western blot and quantification of p16 protein levels in the hippocampus, (*n*=3 animals/group). **b-d** Immunofluorescence staining of p16 co-localization with NeuN, IBA1, and GFAP in four groups, (scale bar=100 µm, 50 µm). Data are expressed as the mean ± SEM. **p* < 0.05.


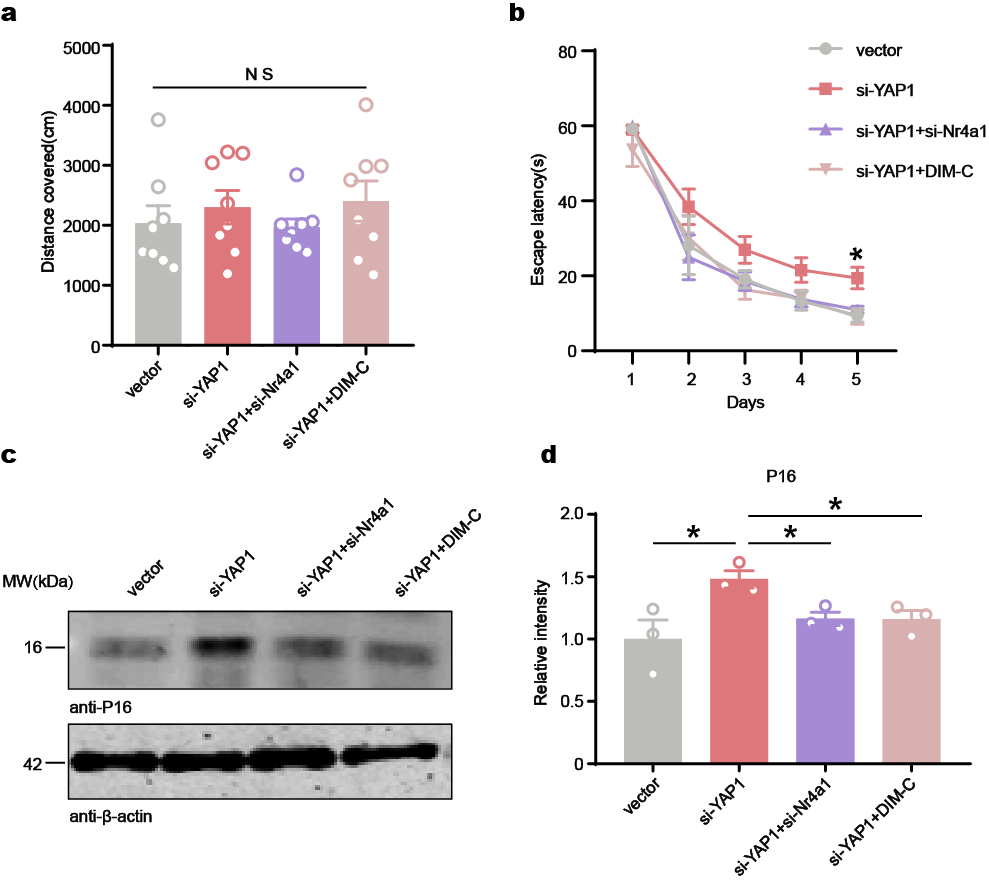


**Figure S5. Nr4a1 was required for YAP1 deficiency to induce cognitive impairment and neuronal senescence**

**a** The total distance traveled by the mice was measured and evaluated in the open-field test, (*n*=8 animals/group). **b** The outcome of the MWM test was measured by the latency required to find the hidden platform from days 1 through 5, (*n*=6-8 animals/group). **c-d** Western blot and quantification of p16 protein levels in the hippocampus, (*n*=3 animals/group). Data are expressed as the mean ± SEM, **P* < 0.05, NS, no significance.
